# Aggressive systemic mastocytosis of colon and lymph node: A case report

**DOI:** 10.1097/MD.0000000000033813

**Published:** 2023-05-26

**Authors:** Shen Xun-Ze, Fang Liu, Chen Lin, Yi-Feng Sun

**Affiliations:** a PET/CT Center, Shaoxing People’s Hospital (Shaoxing Hospital, Zhejiang University School of Medicine), Shaoxing, Zhejiang Province, China; b Department of Pathology, Shaoxing People’s Hospital, Shaoxing, Zhejiang Province, China; c Vascular Surgery, Shaoxing People’s Hospital, Shaoxing, Zhejiang Province, China.

**Keywords:** colon, colonoscopy, computerized tomography, fungal infection, imaging, positron emission tomography, systemic mastocytosis

## Abstract

Mastocytosis is a group of rare neoplastic diseases characterized by monoclonal proliferation of mast cells in the skin or other tissues and organs, including cutaneous mastocytosis and systemic mastocytosis (SM). Mastocytosis can also occur in the gastrointestinal tract, mostly manifested as increased mast cells dispersed in various layers of the intestinal wall; a few may present as polypoid nodules, but rarely as soft tissue mass formation. Pulmonary fungal infections mostly occur in patients with low immune function and have not been reported in the literature as the initial manifestation in patients with mastocytosis. In this case report, we present the enhanced computed tomography (CT), fluorodeoxyglucose (FDG) positron emission tomography/CT, and colonoscopy findings of a pathologically confirmed patient with aggressive SM of the colon and lymph nodes and extensive fungal infection of both lungs.

**Patient concerns:**

A 55-year-old female patient visited our hospital because of repeated cough for more than half a month. Laboratory tests revealed a significantly high CA125 serum level. Chest CT showed multiple plaques and patchy high-density shadows in both lungs, and a small amount of ascites was observed in the lower-level image. Abdominal CT revealed a soft tissue mass with an ill-defined boundary in the lower ascending colon. Whole-body positron emission tomography/CT images showed multiple nodular and patchy density-increasing lesions with significantly increased FDG uptake in both lungs. The wall of the ascending colon in the lower segment was significantly thickened with soft tissue mass formation, and retroperitoneal lymph node enlargement was accompanied by increased uptake of FDG. Colonoscopy revealed a soft tissue mass at the base of the cecum.

**Diagnosis:**

Colonoscopic biopsy was performed and the specimen was diagnosed with mastocytosis. At the same time, a puncture biopsy was also performed on the patient’s lung lesions, and pulmonary cryptococcosis was considered a pathological diagnosis.

**Interventions:**

The patient was in remission after repeated treatment with imatinib and prednisone for 8 months.

**Outcomes:**

In the ninth month, the patient suddenly died of a cerebral hemorrhage.

**Lessons:**

Gastrointestinal involvement due to aggressive SM presents with nonspecific symptoms and different endoscopic and radiologic findings. This is the first report of a single patient with colon SM, retroperitoneal lymph node SM, and extensive fungal infection in both lungs.

## 1. Introduction

Mastocytosis is an uncommon hematopoietic neoplasm characterized by clonal proliferation of atypical mast cells. The clinical manifestations of mastocytosis are heterogeneous, ranging from limited cutaneous involvement to aggressive disease with various levels of extracutaneous multi-organ involvement. Most cases of mastocytosis are indolent, usually cutaneous or indolent systemic mastocytosis (SM). Aggressive SM is a rare and often fatal disease. Gastrointestinal (GI) involvement in aggressive SM presents with nonspecific symptoms and different imaging findings, and the final diagnosis depends on the pathology. Herein, we present a special case of aggressive SM in the colon and lymph nodes with fungal infection of the lungs. To the best of our knowledge, such a case has not yet been reported in literature.

## 2. Case presentation

A 55-year-old female patient visited our hospital because of repeated coughing for more than half a month. The patient had a dry cough with no phlegm and no obvious shortness of breath when using a cold air conditioner more than half a month prior. Subsequently, the above symptoms were aggravated, cough accompanied by a small amount of white sputum, and wheezing, which could be relieved by themselves. The patient had no chills or fever. The local hospital administered cephalosporin antibiotic treatment; however, no obvious effect was observed. The patient was referred to our hospital for further treatment.

Laboratory tests revealed a significantly high CA125 serum level (263.2 U/mL (normal range, 0–35 U/mL), whereas the other tumor markers were within the normal range. Contrast-enhanced chest computed tomography (CT) showed multiple plaques and patchy high-density shadows in both lungs, which were moderately enhanced after contrast medium injection, with blurred edges and bronchial shadows inside. A small amount of ascites was observed in the lower-level image, suggesting that the pulmonary lesions may be multiple metastases from the primary abdominal malignancy.

**Figure 1.**
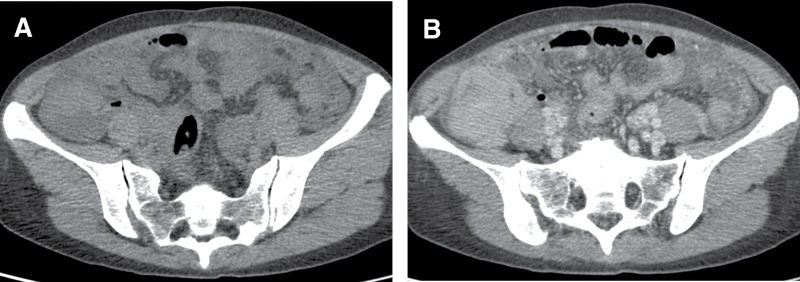
Abdominal contrast-enhanced CT showed a soft tissue mass with ill-defined boundaries in the lower ascending colon, which was significantly enhanced after contrast injection. (A) Plain scan. (B Enhancement scan. CT = computed tomography.

**Figure 2.**
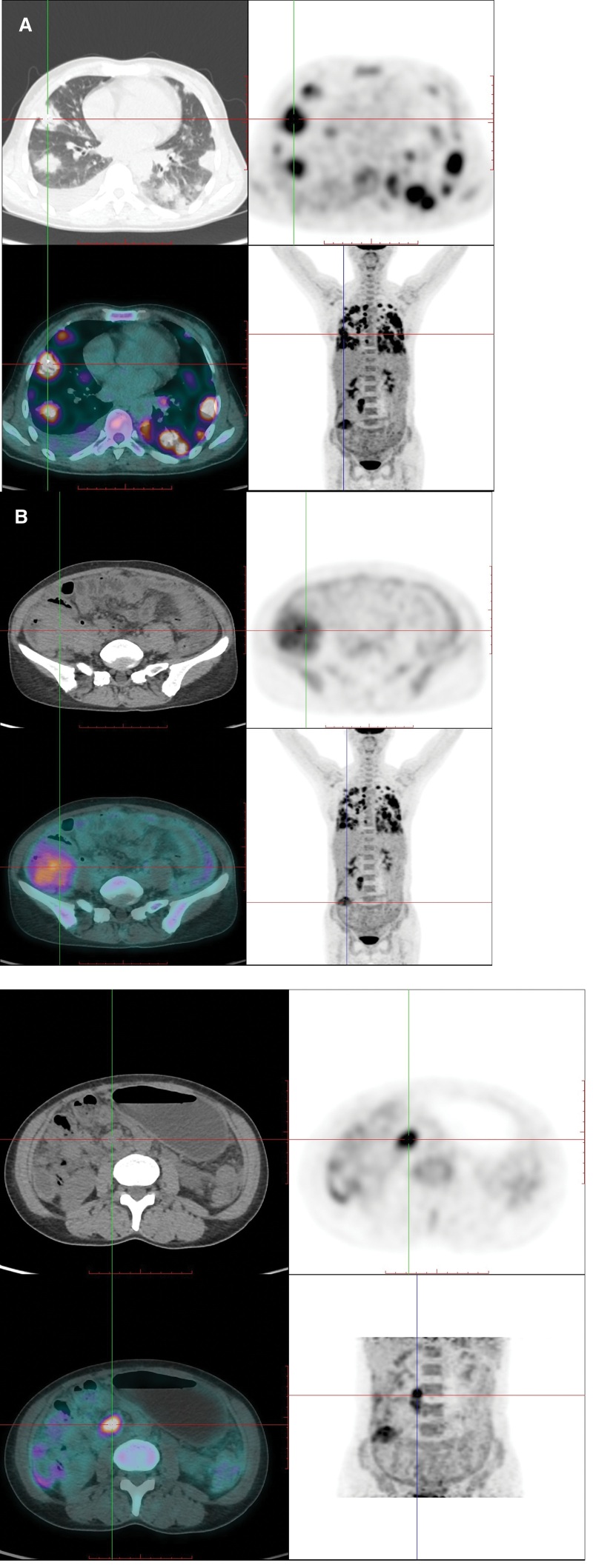
(A) The whole-body PET/CT images showed multiple nodular and patchy density-increasing lesions with significantly increased FDG uptake in both lungs. (B) Soft tissue mass with increased FDG uptake in the lower ascending colon. (C) Retroperitoneal lymph node enlargement with increased FDG uptake on delayed imaging. FDG = fluorodeoxyglucose, PET/CT = positron emission tomography/computed tomography.

**Figure 3.**
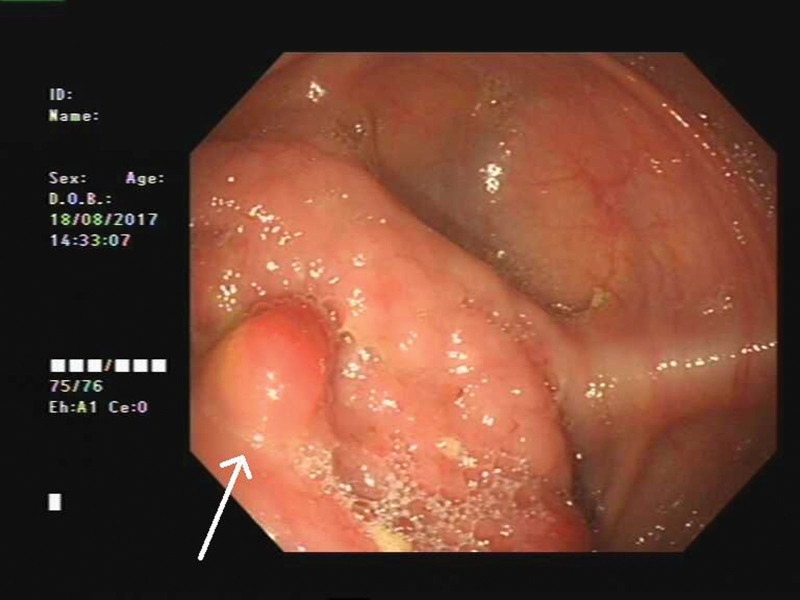
Colonoscopy showed a 3 × 3cm mass in the base of the cecum.

Then, abdominal CT examination was performed, and the results showed a soft tissue mass with an ill-defined boundary in the lower ascending colon, which was significantly enhanced after contrast agent injection (Fig. 1), and malignant tumor was considered. Whole-body positron emission tomography (PET)/CT images showed multiple nodular and patchy density-increasing lesions with significantly increased fluorodeoxyglucose (FDG) uptake in both lungs (Fig. 2A). FDG uptake increased significantly, and the maximum standardized uptake value (SUVmax) was approximately 6.0. The wall of the ascending colon in the lower segment was significantly thickened with soft tissue mass formation (Fig. 2B). The conventional and delayed imaging SUVmax values were approximately 3.6 and 4.4, respectively. Retroperitoneal lymph node enlargement was accompanied by increased uptake of FDG, and SUVmax of routine and delayed imaging were approximately 5.7 and 5.5, respectively (Fig. 2C). A small amount of effusion was observed in the right thoracic, abdominal, and pelvic cavities. Colonoscopy revealed a 3 × 3 cm mass at the base of the cecum, with follicular hyperplasia and bleeding on the surface (Fig. 3). A colonoscopic biopsy was performed.

**Figure 4.**
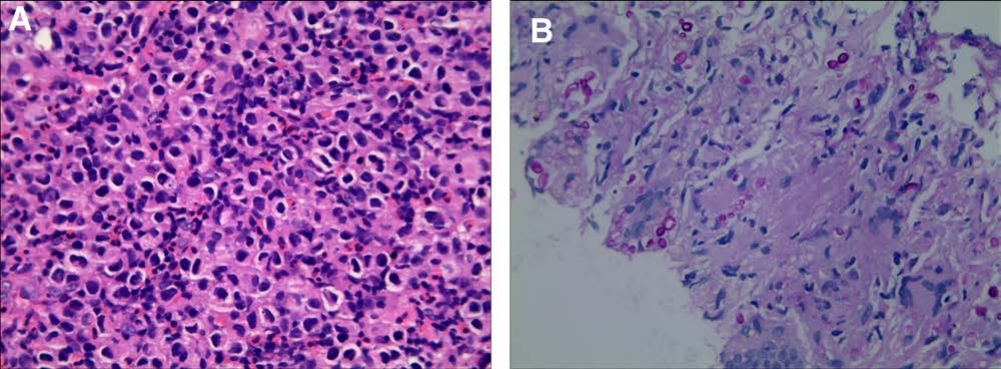
(A) The smears of the colonoscopic specimen showed diffuse infiltration of medium-sized cells in the lamina propria of the colonic mucosa. The cell morphology was relatively uniform, ovoid, or spindle-shaped; the cytoplasm was rich and transparent; the nucleus was round or irregular. (HE, ×400). (B) Biopsy specimens of lung lesions showed the disappearance of normal lung structure, inflammatory cell infiltration, massive histiocytic proliferation, forming multinucleated giant cells, and PAS staining positive bacteria in the cytoplasm of histiocytic cells. (PSA, ×400).

Smears of the colonoscopic specimen showed diffuse infiltration of medium-sized cells in the lamina propria of the colonic mucosa. The cell morphology was relatively uniform, ovoid or spindle-shaped, the cytoplasm was rich and transparent, and the nucleus was round or irregular (Fig. 4A). Immunophenotyping revealed positive staining for CD33, Kit (CD117), and Ki-67 (30%) but negative staining for CD68, S-100, and CK-pan. Therefore, the colonoscopic pathological specimen was diagnosed as a malignant hematopoietic tissue tumor consistent with mastocytosis. At the same time, a puncture biopsy was also performed on the patient’s lung lesions. Pathology showed that normal lung structures disappeared, fibrous hyperplasia, inflammatory cell infiltration, a large number of histiocytic hyperplasia, multinucleated giant cells were formed, PAS staining positive bacteria were observed in the cytoplasm of histiocytic cells, and fungal granuloma (pulmonary cryptococcosis) was considered (Fig. 4B). Thus, the final diagnosis was aggressive mastocytosis of the colon with fungal infection in both lungs.

During the 3-week hospitalization period, the patient developed 3 times of diarrhea. Reviewing her medical history, the patient added that she had also had a few episodes of diarrhea and occasional abdominal pain in the past 6 months.

The patient was repeatedly treated with imatinib and prednisone for 8 months, and the disease was relieved. However, in the ninth month, the patient suddenly died of a cerebral hemorrhage.

Ethical approval was not required as this was a case report of the patient’s clinical information. Written informed consent was obtained from the patient for the publication of this case report and accompanying images.

## 3. Discussion

Mastocytosis is a rare hematopoietic neoplasm characterized by clonal proliferation of atypical mast cells in 1 or more organs.^[1,2]^ Mast cells originate from bone marrow pluripotent stem cells, migrate to specific parts of the body through the blood and lymphatic vessels, and differentiate and mature into mast cells filled with heterochromatic particles. The differentiation and growth of mast cells require a stem cell factor, which expresses c-KIT (stem cell factor receptor) on its surface. Mast cells are mainly distributed in the subepidermis, respiratory tract, GI tract, and urogenital mucosa. Cytoplasmic granules of mast cells contain histamine, heparin, various mucosaccharides, chemokines, and prostaglandins. A variety of immunological and non-immunological stimuli can cause mast cell degranulation and the release of substances, leading to a range of clinical manifestations.

The incidence of mastocytosis is closely related to KIT gene mutation.^[2,3]^ The vast majority of cases are confined to the skin, with approximately 10% of cases involving organs other than the skin, mainly the bone, liver, spleen, GI tract, and lymph nodes.^[1,3]^ The incidence of the disease is 0.01% and increases by 5 to 10 per million per year. Two-thirds of the patients are children, and the disease incidence does not differ in relation to gender, race, environment, or socioeconomic differences^.[4]^ The World Health Organization classification divides mastocytosis into cutaneous mastocytosis, SM, and mast cell sarcoma. Clinically, mastocytosis occurs in both indolent and aggressive forms. Indolent forms, by far the most frequent, include cutaneous mastocytosis, indolent SM, and smoldering SM. Aggressive or high-grade variants of SM, often collectively identified as “advanced SM,” include aggressive SM, SM with associated hematological neoplasm, and MC leukemia.^[5,6]^

Of the extracutaneous sites, the bone marrow is the most frequently involved. GI tract involvement is increasingly recognized because of the increasing awareness among pathologists and physicians involved in the care of patients with mastocytosis and the availability of sensitive and specific immunohistochemical markers to detect neoplastic mast cells.^[7]^ In patients with GI involvement, the symptoms may also be due to mast cells directly infiltrating the mucosa, resulting in malabsorption and/or enhanced local mediator release. The most common symptom of GI mastocytosis is diarrhea, followed by abdominal pain, nausea, weight loss, bloating, vomiting, or reflux. Some patients may also experience intestinal obstructions. The frequency of GI symptoms in patients with SM is estimated to be 60 to 80%.^[1]^ Although our patient was admitted to the hospital due to the clinical manifestations of respiratory diseases, she also suffered from diarrhea 3 times during her hospitalization, and she also had a few bouts of diarrhea and occasional abdominal pain within the previous 6 months.

Histologically, the involved biopsies were characterized by infiltrates of ovoid to spindle-shaped mast cells in aggregates or sheets in the lamina propria, sometimes forming a confluent band underneath the surface epithelium. Colic mucosal mast cell density can also be higher in patients with irritable bowel syndrome, especially in those with diarrhea. The presence of mast cell aggregates or sheets is key to the differential diagnosis. Because of the subtle nature of the infiltrate, immunohistochemistry with KIT (CD117) and CD25 is helpful in the identification of atypical mast cell infiltrates.^[8–11]^

The colon is the most commonly involved site in GI SM, and colonoscopy is the main method used for imaging examination and lesion biopsy. During colonoscopy, SM can present as nodular mucosal lesions, multiple purple-pigmented lesions, polypoid lesions, aphthous ulcers, erosions, granularity, erythema, and even normal-appearing mucosa.^[1,11,12]^ However, the application of CT and PET/CT in GI SM examinations has not yet been reported. In our case, colonoscopy, CT, and PET/CT imaging showed a solitary large soft tissue mass, which has not been previously reported in the existing literature. While colonoscopy can only detect intestinal wall lesions, PET/CT can also detect parenteral lesions by its unique imaging of glucose metabolism, as shown in this case of SM involving an enlarged retroperitoneal hypermetabolic lymph node. In this case, PET/CT also showed extensive fungal infection in both lungs, thus demonstrating the advantage of being “one-stop” examination for PET/CT. Wyre et and Henrichs WD reported a case of SM and eosinophilic granuloma of the lungs.^[13]^ Our patient was admitted with a pulmonary infection and confirmed to have SM by colonoscopy. Extensive fungal infection of both lungs as the initial clinical manifestation in SM patients has not yet been reported in previous literature.

The prognosis and treatment of mastocytosis are closely related to the disease type. For aggressive SM, mast cell leukemia, and mast cell sarcoma, the prognosis is poor and the proliferation of mast cells needs to be blocked. There are no specific treatment methods, and symptomatic treatment is the primary treatment. Radiotherapy or chemotherapy is effective in some patients, and effective treatment with interferon has also been reported.^[14,15]^ In recent years, inhibition of c-KIT tyrosine kinase activity to induce apoptosis in mutant mast cells has emerged as a therapeutic approach. Imatinib used in this case, is one of the commonly used tyrosine kinase inhibitors.^[16]^ Targeted monoclonal antibody therapy and allogeneic hematopoietic stem cell transplantation are new therapeutic methods that are currently being explored.^[17,18]^

In conclusion, SM results from the clonal proliferation of abnormal mast cells in extracutaneous organs. Aggressive SM is a rare and often fatal condition. The colon is the most commonly involved GI SM site, and colonoscopy is the main method for the examination and biopsy of lesions. CT and PET/CT are valuable for detecting extraneous intestinal lesions, especially PET/CT, whose unique imaging of glucose metabolism contributes to the overall diagnosis. GI involvement in aggressive SM presents with nonspecific symptoms and different endoscopic and radiologic findings, while the final diagnosis depends on the pathology. To the best of our knowledge, the combination of colon mass SM, retroperitoneal lymph node SM, and extensive fungal infection of both lungs reported in this paper has not been previously reported.

## Author contributions

**Conceptualization:** Shen Xun-Ze, Yi-Feng Sun.

Formal analysis: Fang Liu.

Resources: Yi-Feng Sun.

Writing – original draft: Shen Xun-Ze, Chen Lin.

Writing – review & editing: Shen Xun-Ze.
